# Treatment of Dysentery by Injections of Nitrate of Silver and Creosote

**Published:** 1850-04

**Authors:** 


### RECORD OF MEDICAL SCIENCE.

### PATHOLOGY AND PRACTICE OF MEDICINE.

*Treatment of Dysentery by injections of Nitrate of Silver and Creosote.* By Professor Flint.—The nitrate of silver, as we know, in analogous instances of inflamed mucous tissue—for example, in conjunctivitis, pharyngitis, &c., exerts a surprising effect in diminishing and arresting inflammatory action. It has been employed, to some extent, in dysentery, and is recommended by some practical writers; but so far as we know, is by no means in common use. In one case we resorted to a solution of the crystals of the nitrate of silver, ten grains to the ounce, with marked benefit. The tenesmus and frequent dejections were relieved in a striking degree, and the discharge of mucus and blood was much diminished. To secure the good effects of this application, it is desirable that the injection be made to pass up the intestine as high as practicable, in order to bring it into contact with a large portion of the inflamed surface. We found the best instrument at hand to be a female bone syringe, with a long pipe, terminating by a perforated bulbous extremity. Perhaps a solution of greater strength might be even more serviceable. The patient was a child four years of age. The application occasioned, apparently, little or no pain; not more than the ordinary enemas of starch and laudanum. Another remedy employed in the same case was a creosote mixture. We have used this remedy in two cases; in one, of chronic dysentery of long standing, the effect was good, but not extraordinary. In the case recently under treatment, we first employed it in connexion with the tincture of opium, and found that the enemas were retained, when with the laudanum alone they were immediately expelled. We employed at first a mixture for each injection (oz. ss.) containing two minims of creosote. Subsequently we employed the creosote alone, increasing the quantity to four minims, and the good effects were striking. The relief of the local symptoms was quite as great as when the opium was given in combination, the disadvantages of the latter being avoided. We feel confident that this will prove a valuable remedy in the dysentery, and we therefore are solicitous that our readers should make trial of it. We do not, of course, suggest these as remedies intended to supersede other therapeutical measures, but only as useful auxiliaries thereto.—*Buffalo Medical Journal.*

